# MRI Analysis of Cardiac Fat, Abdominal Adiposity and Cardiac Function After Vertical Sleeve Gastrectomy in Youth With Type 2 Diabetes

**DOI:** 10.1111/ijpo.70068

**Published:** 2025-11-17

**Authors:** Tyler J. Dobbs, Linnea Gutierrez, Houchun Harry Hu, Kendall S. Hunter, Brian Fonseca, Lorna Browne, David Winter, Melanie G. Cree, Laura Pyle, Jonathan L. Hills‐Dunlap, Jane E. B. Reusch, Megan M. Kelsey, Thomas H. Inge, Petter Bjornstad, Kristen J. Nadeau

**Affiliations:** ^1^ Department of Pediatrics University of Colorado Anschutz Medical Campus and Children's Hospital Colorado Aurora Colorado USA; ^2^ Department of Bioengineering University of Colorado Anschutz Medical Campus and Children's Hospital Colorado Aurora Colorado USA; ^3^ Department of Radiology Mayo Clinic Jacksonville Florida USA; ^4^ Department of Pediatrics University of Washington and Seattle Children's Hospital Seattle WA USA; ^5^ Department of Pediatrics Northwestern University and Lurie Children's Hospital of Chicago Chicago Illinois USA

**Keywords:** adolescent, bariatric surgery, fat distribution, obesity

## Abstract

**Background:**

Adolescents with youth‐onset type 2 diabetes (YO‐T2D) have an increased risk for cardiometabolic complications. The impact of vertical sleeve gastrectomy (VSG) on fat distribution and cardiac morphology/function in YO‐T2D is unknown.

**Objectives:**

To evaluate changes in body composition, abdominal and cardiac fat depots, and cardiometabolic health in adolescents with YO‐T2D undergoing VSG.

**Methods:**

Anthropometrics, labs and imaging were used to assess participants pre‐surgery and 3–12 months post‐surgery. MRI quantified pericardial (PAT), epicardial (EAT), subcutaneous (SAT) and visceral adipose tissue (VAT), hepatic fat fraction (HFF) and cardiac morphology/function. Mixed‐effects models assessed longitudinal changes.

**Results:**

By 12 months, weight decreased from 134.3 ± 5.1 to 103.3 ± 5.2 kg, VAT 1943 ± 148 to 1248 ± 150 cm^3^, HFF 23.2% ± 2.3% to 5.3% ± 2.3% and PAT and EAT by 27% and 33% (all *p* < 0.01). Homeostatic model assessment of insulin resistance (HOMA‐IR) improved (7.8 ± 1.2 to 1.7 ± 1.2 [*p* < 0.01]). BMI, SAT, resting heart rate (RHR), left ventricular (LV) mass and cardiac output (CO) also decreased (all *p* < 0.05). VAT decreases correlated with decreases in HFF (*r* = 0.70, *p* = 0.01), HOMA‐IR (*r* = 0.60, *p* = 0.04) and CO (*r* = 0.70, *p* = 0.03). HFF decreases correlated with decreases in BMI (*r* = 0.70, *p* = 0.03), HOMA‐IR (*r* = 0.90, *p* < 0.001), RHR (*r* = 0.90, *p* = 0.002), CO (*r* = 0.80, *p* = 0.007) and LV mass (*r* = 0.70, *p* = 0.02).

**Conclusions:**

VSG reduces ectopic and regional fat and improves associated insulin sensitivity and cardiac health in adolescents with YO‐T2D.

**Abbreviations**
ALT: alanine aminotransferase
AST: aspartate aminotransferase
CO: cardiac output
CVD: cardiovascular disease
DBP: diastolic blood pressure
EAT: epicardial adipose tissue
EDV: end‐diastolic volume
EF: ejection fraction
ESV: end‐systolic volume
FFM: fat‐free mass
FM: fat mass
GLP‐1RA: glucagon‐like peptide‐one receptor agonist
HFF: hepatic fat fraction
HOMA‐IR: homeostatic model assessment of insulin resistance
IL‐6: interleukin‐6
LV: left ventricle
MAP: mean arterial pressure
MBS: metabolic bariatric surgery
PAT: pericardial adipose tissue
PDFF: proton density fat fraction
RHR: resting heart rate
RV: right ventricle
RYGB: Roux‐en‐Y gastric bypass
SAT: subcutaneous adipose tissue
SBP: systolic blood pressure
SGLT‐2I: sodium‐glucose cotransporter‐2 inhibitor
SV: stroke volume
T2D: type 2 diabetes
TNF‐α: tumour necrosis factor alpha
VAT: visceral adipose tissue
VSG: vertical sleeve gastrectomy

## 1 Introduction

Overweight and obesity pose a major risk for many chronic diseases including cardiovascular disease (CVD) and type 2 diabetes (T2D). In the USA, one in five adolescents is estimated to have obesity [1], and as a result, rates of obesity‐related comorbidities such as T2D and early CVD have risen in parallel [2]. Unlike adult‐onset T2D, youth‐onset T2D appears to be a more aggressive disease, with faster progression of β‐cell dysfunction and insulin resistance, leading to earlier onset of complications, including CVD [3]. The Restoring Insulin Secretion (RISE) study demonstrated significant differences in the pathophysiology of T2D between youth and adults, finding that youth exhibit more severe insulin resistance and a more rapid decline in β‐cell function than adults [4, 5]. These findings underscore the distinct challenges in managing youth‐onset T2D and the urgent need for targeted and durable interventions tailored to this underrepresented and high‐risk population.

Adipose tissue plays a central role in the pathophysiology of T2D, with ectopic fat depots contributing to systemic inflammation and metabolic dysregulation [6]. Visceral adipose tissue (VAT) is more strongly associated with adverse cardiometabolic outcomes than subcutaneous adipose tissue (SAT), including increased risk for insulin resistance, T2D, dyslipidemia and CVD [7]. Cardiac fat depots measurable by MRI include two distinct fat depots: epicardial adipose tissue (EAT) and paracardial adipose tissue, which make up the total cardiac fat called pericardial adipose tissue (PAT). They differ from other fat depots, not only in their anatomy but also in their endocrine functions and embryology [8]. Epicardial fat is located between the outer wall of the myocardium and the visceral layer of the pericardium. Pericardial fat is anterior to the epicardial fat and therefore located between visceral and parietal pericardium [6, 8]. Due to its anatomical proximity to the heart and its vascular supply originating from the coronary circulation, it is reasonable to expect excess EAT to have negative consequences on cardiac health [6].

In adults, metabolic bariatric surgery (MBS) induces significant and durable weight loss of ~28.4% after 7 years [9], and profoundly alters fat distribution, particularly reductions in VAT and EAT [10]. These changes are associated with improvements in cardiometabolic risk factors including hypertension, insulin resistance and liver enzyme elevations [11, 12]. MBS in adolescents elicits significant weight loss, but fewer data exist on its impact on cardiovascular health. The Teen‐Longitudinal Assessment of Bariatric Surgery (Teen‐LABS) study recently published 10‐year outcomes demonstrating sustainable cardiometabolic improvements of MBS in youth, including durable weight loss and improvements in glycemic control and comorbidities such as hypertension and dyslipidemia [13]. However, while the two largest cohort studies of bariatric surgery in youth, Teen‐LABS [13] and the Swedish Obesity Study [14], provide valuable insight into the metabolic and health benefits of MBS in youth, they did not specifically investigate changes in regional fat distribution, such as EAT, PAT, VAT, or SAT or cardiac morphology or function. Moreover, most of the youth in these cohorts underwent the preferred procedure of that time, Roux‐en‐Y gastric bypass (RYGB), whereas vertical sleeve gastrectomy (VSG) currently accounts for > 90% of MBS in youth [15]. Additionally, fat distribution changes following MBS may more accurately reflect improvements in long‐term cardiovascular risk, particularly in youth. Studies in adults have linked reductions in ectopic fat depots to improved cardiac geometry and metabolic outcomes, but similar data are lacking in younger populations [16].

The Impact of Metabolic surgery on Pancreatic, Renal and cardiovascular health in youth with T2D (IMPROVE‐T2D) study is a clinical trial (NCT03620773) to determine the effect of MBS in the form of VSG on cardiometabolic health. IMPROVE‐T2D showed significant early improvements in body weight, body fat, percent insulin sensitivity and secretion and glycemic control at 3 months post VSG, with most participants no longer requiring diabetes medications by 3 months following VSG [17]. Here we assess the impact of MBS on cardiac and abdominal fat depots and cardiac morphology and function in youth with T2D, using advanced MRI techniques, three and 12 months after VSG. By focusing on this unique population, we seek to provide novel insights into the interplay between adipose tissue distribution, metabolic outcomes and cardiovascular health.

## 2 Material and Methods

### 2.1 Participants

Sixteen adolescents between the ages of 14–19 years with confirmed antibody‐negative youth‐onset T2D and severe obesity (BMI ≥ 35 kg/m^2^) were recruited from the Paediatric MBS clinic at Children's Hospital Colorado. The study and recruitment criteria were approved by the Colorado Multiple Institutional Review Board (COMIRB Approval #18‐0704). This analysis represents a pre‐planned secondary analysis of a prospective study designed to evaluate metabolic and cardiovascular changes following VSG.

Written informed consent was obtained by qualified research personnel for participants ages 18 years or older and assent for participants below 18 years of age in addition to the parent(s) or legal guardian providing consent. Detailed methods and participants have been previously described [17]. Briefly, inclusion criteria included confirmed antibody‐negative youth‐onset T2D (negative for insulin, glutamic acid decarboxylase, islet‐cell and zinc transporter antibodies) and severe obesity, scheduled for VSG and exclusions included diabetes diagnosis after age 18, prepubertal status, anaemia, pregnancy or breastfeeding or recent diabetic ketoacidosis. Prior to the initial study visit after screening, participants were asked to refrain from strenuous physical activity for 3 days prior to the visit to reduce the acute effects on insulin sensitivity and glucose levels. Metformin and sodium‐glucose cotransporter 2 inhibitors (SGLT‐2i) were discontinued 72 h, GLP‐1 receptor analogues (GLP‐1RAs) 1 week, and long‐acting insulin 24 h prior to their study visit if applicable. Participants arrived in the AM fasting to undergo study procedures. The identical study visit occurred at three visits: pre‐surgery, 3 months post‐surgery and 12 months post‐surgery. Baseline assessments were conducted a median of 34 days (approximately 4.8 weeks; range 1–184 days, 0–26 weeks) prior to VSG.

### 2.2 Clinical Visit Measurements

Height was measured to the nearest 0.1 cm by a standard stadiometer. Weight was measured using a digital scale and rounded to the nearest 0.1 kg. BMI and BMI percentiles were calculated. Waist and hip circumference were taken using a flexible tape measure to the nearest 0.1 cm. Waist circumference was taken at the narrowest part of the waist above the umbilicus and below the xiphoid process. Hip circumference was taken at the maximal girth of the hip, around the buttocks above the gluteal fold. Measurements were taken twice and then averaged. Body composition was measured with air‐displacement plethysmography (BodPod, Cosmed Inc., Rome, Italy) to determine total body fat percent, fat mass (FM) and fat‐free mass (FFM). Systolic (SBP) and diastolic blood pressure (DBP) were measured twice manually after 5 min of rest using a stethoscope and sphygmomanometer and the average of the two readings was recorded and used to calculate mean arterial pressure (MAP) as MAP = DBP + 1/3(SBP − DBP). Resting heart rate (RHR) was manually counted for 60 s after 5 min of rest. A fasting blood draw was taken for glucose, insulin, lipid panel, alanine aminotransferase (ALT) and aspartate aminotransferase (AST) using standard clinical methods and analysed at the Colorado Clinical and Translational Sciences Research Centre Laboratory. HbA1c was measured using the potassium ferricyanide standardised method (Siemens DCA Vantage). Insulin sensitivity was estimated using the homeostatic model assessment for insulin resistance (HOMA‐IR) and calculated as (fasting insulin [μU/mL] × fasting glucose [mmol/L])/22.5.

### 2.3 MRI Measurements

#### 2.3.1 Cardiac MRI Acquisition and Adipose Tissue Analysis

Short axis cardiac images were acquired using a 1.5T Philips MRI system (Ingenia, R5.7, Philips Healthcare, The Netherlands). A multi‐channel cardiac coil array was used and placed anteriorly on the participant. A 2D balanced gradient‐recalled‐echo pulse sequence was used, along with retrospective electrocardiogram gating. A total of 14 slices were acquired with 8–9 mm slice thickness, with an in‐plane spatial resolution of 1.0–1.6 mm × 1.0–1.6 mm.

**FIGURE 1:**
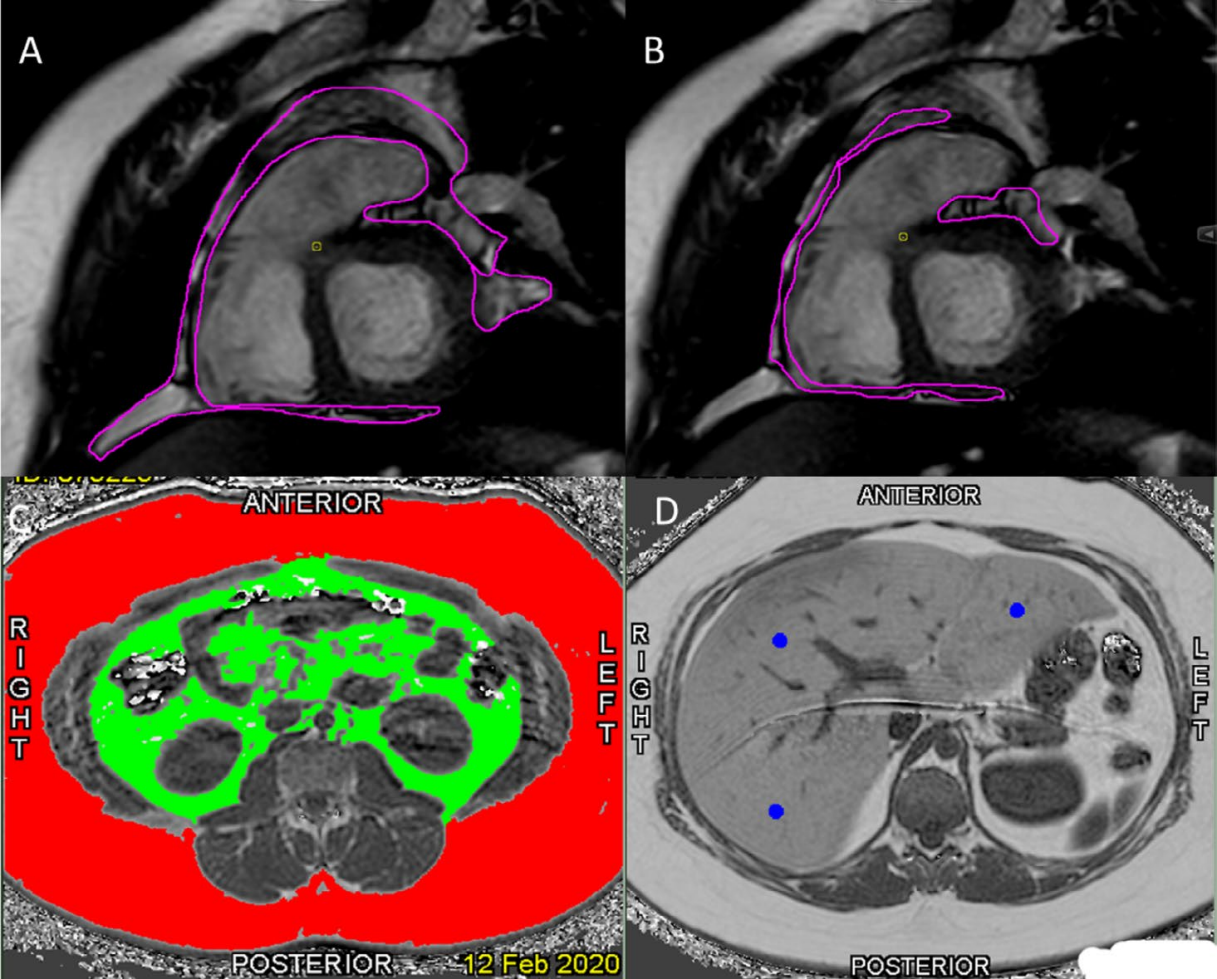
Representative MRI images illustrating segmentation of adipose tissue compartments and hepatic fat fraction. (A) Pericardial adipose tissue is outlined in purple and was contoured separately from the myocardial surface to the external border of the paracardial adipose tissue. (B) Epicardial adipose tissue is delineated in purple and lies between the myocardial surface and the visceral layer of the pericardium. (C) Subcutaneous adipose tissue (red) and visceral adipose tissue (green) are segmented in an abdominal cross‐section slice. (D) Hepatic fat fraction is assessed by averaging regions of interest (blue). These segmentations were performed using automated and manual contouring techniques to quantify adipose tissue distribution.

Epicardial and pericardial adipose tissue compartments were manually contoured in end‐diastole when the ventricular blood pool cavity is the largest and the atrioventricular valves are closed. Analysis was performed using dedicated cardiovascular MRI software (cvi42, v.5.11.2; Circle Cardiovascular Imaging, Calgary, Canada). We adapted previous methods for standard operating procedures to quantify cardiac adipose tissue [18]. Briefly, cardiac fat was manually delineated on the same short axis stack images from the atrioventricular valve annuli to the apex. EAT was defined as the volume between the outer wall of the myocardium and the epicardium. PAT contains all adipose tissue surrounding the heart and was contoured separately from the myocardial surface to the external border of the paracardial adipose tissue (Figure 1A). EAT was measured by contouring the fat depot that lies between the myocardial surface and the visceral layer of the pericardium (Figure 1B). Adipose tissue volume was reported in cm^3^.

#### 2.3.2 Cardiac Morphology and Function Analysis

Cardiac morphology and function were measured on a short axis cine stack covering both ventricles, using Circle CVI 42 following standard methodology [19]. Automated contouring was used with manual review and correction as necessary. Rounded contours were used, including trabeculae and papillary muscles in the ventricular volume. The results were subject to quality control with less than 10% variance between the right and left ventricular stroke volumes (SVs).

#### 2.3.3 Abdominal MRI Acquisition and Adipose Tissue Analysis

Abdominal transverse (axial) images were acquired using a 1.5T Philips MRI system (Ingenia, R5.7, Philips Healthcare, The Netherlands). A multi‐channel torso coil array was used and placed anteriorly on the participant. A 3D multi‐breath‐hold chemical‐shift‐encoded water‐fat separation pulse sequence was used (i.e., mDIXON‐Quant on Philips MRI systems). The pulse sequence acquires multi‐echo data and reconstructs water‐only images, fat‐only images and proton‐density‐fat‐fraction (PDFF) maps. The PDFF maps provide a voxel‐wise representation of the underlying fat content and can be used to quantify adipose tissue depot volumes and organ fat content. A total of 130 slices were acquired with either 5‐ or 10‐mm slice thickness, with an in‐plane spatial resolution range of 1.2–1.7 mm × 1.2–1.7 mm.

SAT, VAT and hepatic fat fraction (HFF) were measured using commercially available software (SliceOmatic, Tomovision Inc., Quebec, Canada). Briefly, manual segmentation was performed to delineate the subcutaneous (red) and visceral (green) adipose tissue depots using the morphology and region growing function [20] (Figure 1C). Slices were continually segmented starting at the first slice in which the kidney and adrenal glands were visible and continued superiorly past the pancreas, covering approximately 25 cm in distance for each participant. To estimate HFF, three regions of interest were drawn on the right and left lobes of the liver and an average PDFF was reported (Figure 1D).

### 2.4 Statistical Analysis

Participant characteristics were summarised as mean and standard deviation (SD) or as counts and percentages. Repeated measures mixed‐effects models with a compound‐symmetric correlation structure were used to examine changes from pre‐surgery to 3 and 12 months post‐surgery and summarised as mean and standard error (SE). Unadjusted models and models adjusted for weight loss were performed. Estimated marginal means were calculated at each visit. Pearson's correlation analysis was used to assess the relationships between continuous variables. A *p*‐value of < 0.05 was considered statistically significant. Tukey's Honestly Significant Difference adjustment was used to correct for multiple comparison of timepoint means for each outcome. No correction was made for testing multiple outcomes, as these analyses are considered hypothesis‐generating. Analyses were performed using R version 4.0 (R Core Team, Vienna, Austria) and the correlation analysis was performed, and figures created using GraphPad Prism version 10.2.1 (GraphPad Software, Boston, USA).

## 3 Results

### 3.1 Baseline Characteristics

Participants had a mean age of 16.6 ± 1.9 years, were 56.2% female, and were 68.8% Hispanic White, 18.8% Non‐Hispanic White and 12.5% Non‐Hispanic Black (Table 1). They had an average T2D duration of 17.6 ± 13.1 months with an average age at diagnosis of 15.9 ± 1.4 years. All participants were on diabetes medications (metformin, SGLT2i, GLP‐1RA and/or insulin, Table 1), with a majority on multiple agents (seven on one medication, seven on two medications and two on three medications).

**TABLE 1:** Participant characteristics at baseline.

Variable	Baseline
*n*	16
Age (years)	16.4 ± 1.7
Sex (F/M)	9/7
Race/Ethnicity (*n* [%])	
Hispanic or Latino	11 (68.8%)
Non‐Hispanic Black	2 (12.5%)
Non‐Hispanic White	3 (18.8%)
Diabetes duration (months)	17.3 ± 13.1
Age at diagnosis of diabetes (year)	15.4 ± 1.7
Metformin use (*n* [%])	15 (94%)
SGLT‐2i use (*n* [%])	6 (37.5%)
GLP‐1RA use (*n* [%])	3 (18.8%)
Insulin use (*n* [%])	2 (12.5%)

*Note:* Continuous variables are reported as mean ± SD or *n* (%).

Abbreviations: GLP‐1RA, glucagon‐like peptide‐1 receptor agonists; SGLT‐2i, sodium‐glucose cotransporter 2 inhibitor.

### 3.2 Anthropomorphic and Clinical Changes Following MBS

Body weight, BMI and waist and hip circumference decreased significantly by 3 months and continued to decline through 12 months (*p* < 0.01) (Table 2). On average, participants lost 18% of their initial body weight by 3 months and over 23% by 12 months. FM decreased significantly at both timepoints (both *p* > 0.05), while FFM declined modestly and non‐significantly. Improvements in glycemic control were rapid and sustained with HbA1c being significantly lower by 3 months (*p* = 0.01) and sustained at 12 months (*p* < 0.01). 86% of participants no longer required anti‐diabetic medications at 12 months. Insulin resistance measured by HOMA‐IR also showed a marked decrease at 3 months (*p* = 0.01), with further reductions by 12 months (*p* < 0.01).

**TABLE 2:** Anthropomorphic and clinical changes following surgery.

Variable	Pre‐surgery	3 months post‐surgery	12 months post‐surgery	*p* (Pre vs. 3 months)	*p* (Pre vs. 12 months)	*p* (3 months vs. 12 months)
Height (cm)	168.9 ± 7	168.3 ± 7.3	168.3 ± 6.8	0.75	0.75	0.90
Weight (kg)	134.3 ± 5.1	109.7 ± 5.2	103.3 ± 5.2	< 0.01	< 0.01	0.21
BMI (kg/m^2^)	47.0 ± 2	38.5 ± 2	36.2 ± 2	< 0.01	< 0.01	0.21
BMI % of the 95th %ile	161.3 ± 29	130.4 ± 21.1	123.4 ± 21.4	< 0.01	< 0.01	0.21
Waist circumference (cm)	133.6 ± 3.4	117.6 ± 3.4	112.4 ± 3.4	< 0.01	< 0.01	0.14
Hip circumference (cm)	131.2 ± 3	118.7 ± 3	115.2 ± 3	< 0.01	< 0.01	0.31
HbA1c (%)	6.6 ± 0.2	5.7 ± 0.2	5.7 ± 0.2	0.01	0.004	0.94
HOMA‐IR	7.8 ± 1.2	2.2 ± 1.2	1.7 ± 1.2	0.01	< 0.01	0.97
Resting heart rate (bpm)	94 ± 4	75 ± 5	71 ± 5	0.05	0.01	0.69
SBP (mmHg)	130 ± 3	122 ± 3	117 ± 3	0.24	0.01	0.29
DBP (mmHg)	74 ± 2	68 ± 2	67 ± 2	0.19	0.08	0.87
MAP (mmHg)	93 ± 2	86 ± 2	84 ± 2	0.13	0.01	0.55
Total cholesterol (mg/dL)	176 ± 7	142 ± 7	148 ± 7	< 0.01	< 0.01	0.78
LDL‐C (mg/dL)	119 ± 8	97 ± 8	94 ± 8	0.01	< 0.01	0.89
HDL‐C (mg/dL)	34 ± 2	34 ± 2	38 ± 2	0.99	0.03	0.04
Triglycerides (mg/dL)	251 ± 36	119 ± 39	102 ± 39	0.03	0.01	0.92
ALT (U/L)	60 ± 6	28 ± 7	23 ± 6	0.01	< 0.01	0.86
AST (U/L)	55 ± 45	34 ± 5	27 ± 5	0.03	< 0.01	0.69
Body fat (%)	49.1 ± 1.6	45.1 ± 1.7	42.6 ± 1.7	0.05	< 0.01	0.21
Fat mass (kg)	66.9 ± 4.2	53.9 ± 4.5	50.1 ± 4.7	0.02	0.01	0.57
Fat‐free mass (kg)	66.4 ± 3.2	62.3 ± 3.4	62.5 ± 3.5	0.16	0.41	0.96
PAT (cm^3^)	105.2 ± 8.4	83.4 ± 8.4	77 ± 8.6	< 0.01	< 0.01	0.49
EAT (cm^3^)	48.7 ± 2.4	37.8 ± 2.4	32.2 ± 2.6	< 0.01	< 0.01	0.16
SAT (cm^3^)	5158 ± 479	3385 ± 474	4056 ± 487	< 0.01	0.03	0.21
VAT (cm^3^)	1943 ± 148	1423 ± 146	1248 ± 150	< 0.01	< 0.01	0.11
HFF (%)	23.2 ± 2.3	8 ± 2.3	5.3 ± 2.3	< 0.01	< 0.01	0.42

*Note:* Continuous variables are reported as mean ± standard error. Comparisons between time points were tested using repeated measures mixed‐effects models while adjusting for weight loss, with *p*‐values indicating statistical significance for pre‐surgery vs. 3 months (pre vs. 3 months), pre‐surgery vs. 12 months (pre vs. 12 months) and 3 months vs. 12 months (3 months vs. 12 months). A *p*‐value < 0.05 was considered statistically significant and was adjusted for weight loss.

Abbreviations: EAT, epicardial adipose tissue; HFF, hepatic fat fraction; PAT, pericardial adipose tissue; SAT, subcutaneous adipose tissue; VAT, visceral adipose tissue.

SBP and MAP declined significantly by 12 months (*p* = 0.01). RHR decreased significantly by 3 months (*p* = 0.05) and remained reduced at 12 months (*p* = 0.01). DBP did not change. Lipid profiles improved over time with total cholesterol, LDL cholesterol and triglycerides declining significantly by 3 months (all *p* < 0.05) and remained lower at 12 months (*p* < 0.01). HDL cholesterol was unchanged at 3 months but was significantly increased by 12 months (*p* = 0.03). ALT and AST decreased significantly at both timepoints (both *p* < 0.05).

### 3.3 Fat Distribution Following MBS

**FIGURE 2:**
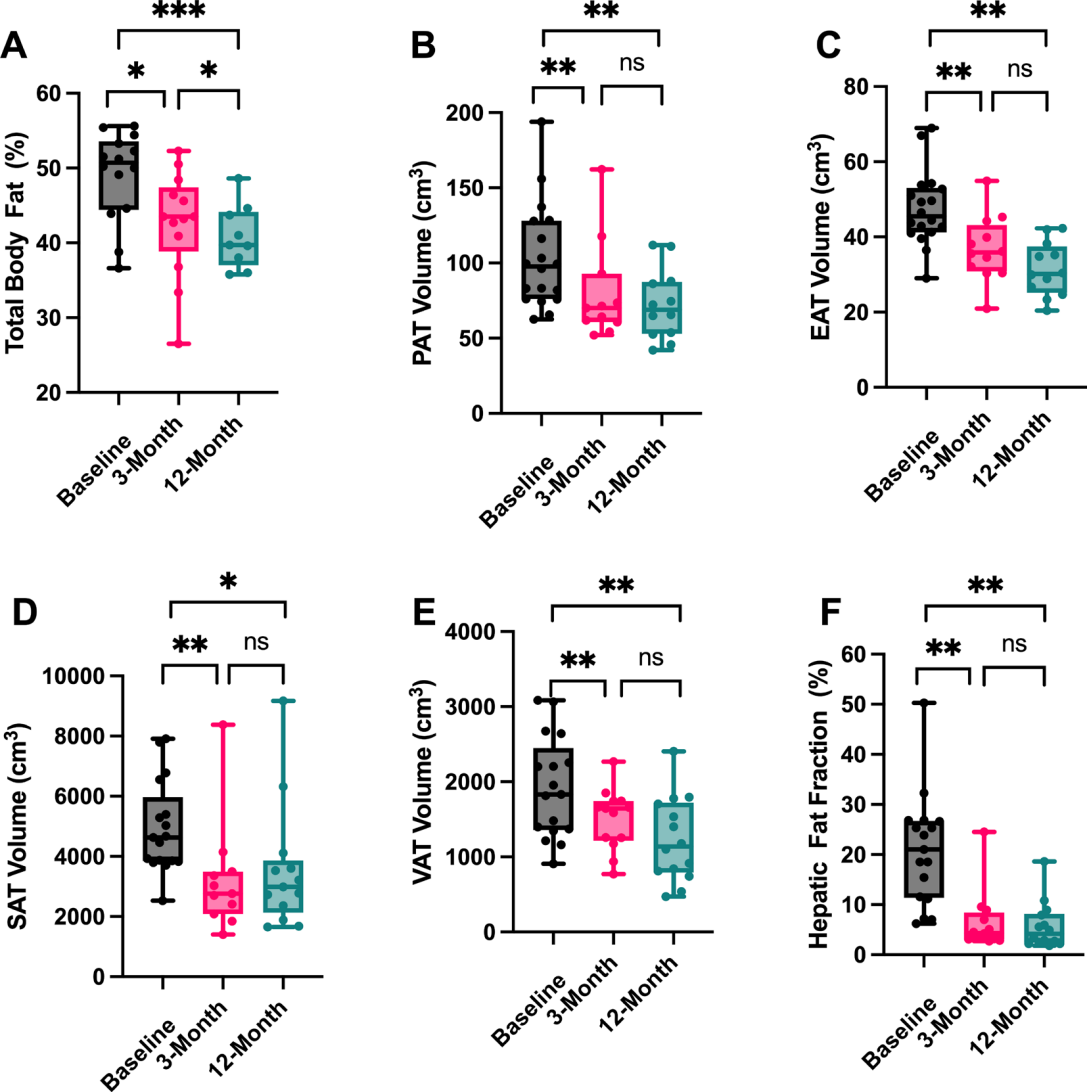
Effect of VSG on fat distribution. Body fat percent (A), PAT (B), EAT (C), SAT (D), VAT (E) and HFF (F) changes at 3‐ and 12‐months post‐surgery. Data are represented with box and whisker plots showing median and all data points min to max. Statistical significance: *p < 0.05, **p < 0.01; and are adjusted for weight loss. EAT, epicardial adipose tissue; HFF, hepatic fat fraction; PAT, pericardial adipose tissue; SAT, subcutaneous adipose tissue; VAT, visceral adipose tissue.

**FIGURE 3:**
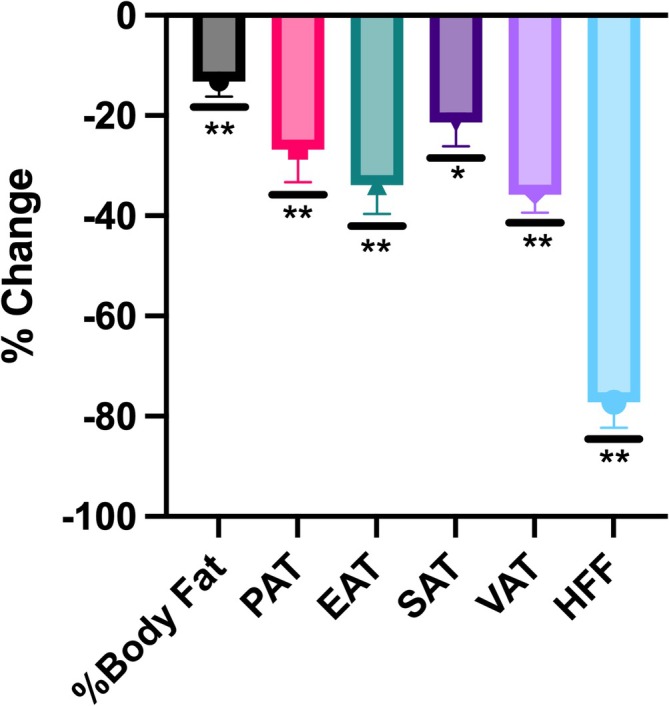
Percent change in MRI analysed fat depots 12 months post‐VSG. Data are presented as mean ± SE. Fat depots analysed include total body fat, pericardial adipose tissue (PAT), epicardial adipose tissue (EAT), subcutaneous adipose tissue (SAT), visceral adipose tissue (VAT) and hepatic fat fraction (HFF). All fat depots demonstrated a reduction, with the greatest percentage decrease observed in HFF. Statistical significance *p < 0.05, **p < 0.01 for percent change from baseline to 12 months and are adjusted for weight loss.

Reductions were observed across all regional fat depots with most occurring within 3 months and sustained thereafter. Total body fat percentage decreased significantly from baseline to 3 months (*p* = 0.05) and continued to decline by 12 months post‐surgery (*p* = 0.01) (Figure 2A). PAT and EAT volumes decreased significantly at both follow‐up timepoints (both *p* < 0.01), with relative reductions exceeding 25% (Figures 2B,C and 3). SAT and VAT also showed significant declines over time, with VAT reductions of 27% by 3 months (*p* < 0.01) and reaching over 30% by 12 months (*p* < 0.01) and SAT reductions of 34% by 3 months (*p* < 0.01) and 21% at 12 months (*p* = 0.03) (Figures 2D,E and 3). HFF decreased markedly from 23.2% ± 2.3% at baseline to 8% ± 2.3% at 3 months and further reduced to 5.3% ± 2.3% at 12 months, corresponding to a relative 66% reduction at 3 months (*p* < 0.01) and over a 75% reduction observed by 12 months (*p* < 0.01) (Figures 2F and 3).

Both changes in VAT and HFF were strongly associated with clinical outcomes. The percent change in VAT was positively correlated with the percent changes in SAT (*r* = 0.60, *p* = 0.03), HFF (*r* = 0.70, *p* = 0.01), HOMA‐IR (*r* = 0.60, *p* = 0.04) and LVCO (*r* = 0.70, *p* = 0.03). Similarly, the percent change in HFF was positively correlated with percent changes in SAT (*r* = 0.70, *p* = 0.01), VAT (*r* = 0.70, *p* = 0.01), BMI (*r* = 0.70, *p* = 0.03), HOMA‐IR (*r* = 0.90, *p* < 0.001), RHR (*r* = 0.90, *p* = 0.002), LVCO (*r* = 0.80, *p* = 0.007) and LV mass (*r* = 0.70, *p* = 0.02).

There were no significant differences between three and 12 months in any variable assessed, other than HDL‐C increasing from three to 12 months (*p* = 0.03) (Table 2).

### 3.4 Cardiac Morphology and Function Following MBS

Cardiovascular morphology and function findings are summarised in Table 3. For left ventricle (LV) parameters, LV end‐diastolic volume (LVEDV) remained stable across all time points, while LV end‐systolic volume (LVESV) showed a trend towards an increase at 3 months but was not significantly different from baseline or 12‐month values. LV ejection fraction (LVEF) was reduced at 3 months (*p* = 0.04) but remained within the normal range and returned to near‐baseline levels by 12 months. LV cardiac output (LVCO) decreased significantly at 3 months (< 0.01) and remained reduced at 12 months (*p* < 0.01), while LV stroke volume (LVSV) remained unchanged. LV mass declined significantly at both follow‐up timepoints (both *p* < 0.05). For right ventricle (RV) parameters, RVEDV, RVESV, RVEF and RVSV were unchanged throughout follow‐up. However, RVCO declined significantly at three (*p* = 0.01) and 12 months (*p* = 0.02), mirroring changes in LVCO.

**TABLE 3:** Cardiac geometry changes following VSG.

Variable	Pre‐surgery	3 month post‐surgery	12 month post‐surgery	*p* (Pre vs. 3 months)	*p* (Pre vs. 12 months)	*p* (3 months vs. 12 months)
LVEDV (mL)	166.6 ± 7	170 ± 7	165 ± 7	0.92	0.99	0.74
LVESV (mL)	70.1 ± 4	76.6 ± 4	73 ± 4.1	0.07	0.53	0.33
LV SV (mL)	96.9 ± 4.5	93.1 ± 4.5	91.6 ± 4.9	0.69	0.72	0.95
LV EF (%)	58.3 ± 1.4	54.8 ± 1.4	56.3 ± 1.5	0.04	0.37	0.57
LV mass (g)	111.4 ± 5.8	100.9 ± 5.8	97.1 ± 5.9	0.02	< 0.01	0.46
LV CO (L/min)	6.7 ± 0.3	4.9 ± 0.3	5 ± 0.3	< 0.01	< 0.01	0.99
RVEDV (mL)	185.6 ± 9.6	178.3 ± 9.6	176.1 ± 10.2	0.74	0.67	0.99
RVESV (mL)	92.5 ± 5.7	88.8 ± 5.7	88.1 ± 5.9	0.77	0.67	0.98
RV SV (mL)	93.4 ± 5.2	89.3 ± 5.2	88.2 ± 5.7	0.71	0.82	0.98
RV EF (%)	50.1 ± 1.6	50.2 ± 1.6	50.7 ± 1.7	0.99	0.91	0.92
RVCO (L/min)	6.5 ± 0.3	4.8 ± 0.3	4.9 ± 0.4	0.01	0.02	0.84

*Note:* Continuous variables are reported as mean ± standard error. Comparisons between time points were tested using repeated measures mixed‐effects models while adjusting for weight loss, with *p*‐values indicating statistical significance for pre‐surgery vs. 3 months (pre vs. 3 months), pre‐surgery vs. 12 months (pre vs. 12 months) and 3 months vs. 12 months (3 months vs. 12 months). A *p*‐value < 0.05 was considered statistically significant and was adjusted for weight loss.

Abbreviations: EDV, end‐diastolic volume; EF, ejection fraction; ESV, end‐systolic volume; LV, left ventricle; RV, right ventricle; SV, stroke volume.

## 4 Discussion

Youth with T2D differ from their adult counterparts not only in the severity of insulin resistance and hyperinsulinemia, but also in their patterns of adipose tissue deposition and response to therapeutic interventions [21]. Here we show the novel findings that VSG in youth‐onset T2D leads to significant and sustained reductions at 1 year in body weight, SAT and ectopic adipose tissue and cardiometabolic risk factors, with peak improvements observed by 3 months post‐op, despite not having yet reached their BMI nadir. These reductions were accompanied by improvements in insulin sensitivity and cardiovascular parameters, including lower triglycerides, total cholesterol, LDL‐C, RHR, BP and LV mass and higher HDL‐C. Collectively, these findings underscore the rapid and durable metabolic and cardiovascular benefits of VSG in youth‐onset T2D.

### 4.1 Weight Loss Outcomes

One year after VSG, our cohort showed a relative reduction in weight of 23%, similar to other prospective adolescent MBS studies with at least 1 year follow‐up (approximately 15%–30% relative reduction) [14, 22, 23, 24]. Our findings in respect to weight loss are lower than some adolescent studies; however direct comparison is limited by differences in study design, including type of surgery and participant characteristics (with vs. without T2D). For example, Olbers et al. reported a 27.8% reduction at 5 years in an adolescent cohort undergoing RYGB, but only 4% had T2D [14]. Alqahtani et al. [23] reported a 32.4% reduction at over 7 years in a large adolescent cohort undergoing VSG (*n* = 2504), but only 10.5% had T2D. Finally, the Teen‐LABS study had a mixed RYGB and VSG cohort that showed a 27% reduction (28% with RYGB, 26% with VSG) at 3 years, but included only three participants with T2D undergoing VSG [24]. Given potential differences in weight loss responses in youth with T2D, yet significant improvements in glycemia, high rates of diabetes remission and cardiometabolic improvements we report [13], future work should prioritise refined cardiometabolic endpoints for choosing surgical candidates and assessing the success of surgery, rather than weight loss alone.

### 4.2 Fat Distribution Changes After VSG

In the current study, we observed the novel finding of significant reductions in SAT, VAT, HFF, EAT and PAT within the first 3 months after VSG, with minimal additional change thereafter, indicating that changes in fat distribution occur early and likely do not require maximal weight loss. The reductions in HFF we found in particular support the metabolic benefits of VSG in youth‐onset T2D, as excess hepatic fat has been strongly correlated with increased cardiovascular and diabetes risk and altered cardiac hemodynamics [25]. Importantly, we found a robust correlation between reductions in HFF and improvements in insulin sensitivity (*r* = 0.90), as well as with VAT, RHR, CO and LV mass. The decline in HFF in this study also aligns with prior evidence in adults showing that hepatic fat loss following MBS is linked to improvements in insulin sensitivity, lipid metabolism and overall cardiovascular function [25, 26]. Only one prior study to date has reported HFF outcomes in adolescents after VSG, showing a seven‐percentage point reduction (from 9.5% to 3%), corresponding to a 68% relative decrease over 6 months [27]. In comparison, participants in our cohort experienced a 17.7 percentage point reduction in HFF (from 23.0% to 5.3%), corresponding to a 77% relative decrease at 12 months post‐surgery. The greater magnitude of HFF reduction in our study may reflect the higher baseline levels of hepatic fat among youth with T2D compared to adolescents without diabetes in the previous study or due to longer follow‐up.

The mechanisms involved in the reduction in HFF following MBS are complex and likely interconnected including reductions in insulin resistance, inflammation and hepatic free fatty acid delivery (from reduced dietary intake and improved suppression of lipolysis secondary to improved adipocyte insulin sensitivity) [16]. Importantly, we also saw significant reductions in ALT and AST in parallel with the loss of HFF, implying that hepatic inflammation also improved, although we lacked repeat liver biopsies in all participants to confirm this histologically. Changes in incretin hormones including GLP‐1, glucose‐dependent insulinotropic polypeptide and peptide YY, as well as bile acid signalling and gut microbiome, and/or improved hepatic mitochondrial function and lipid oxidation, are other potential contributors to the reduction in HFF and sustained cardiometabolic benefits [28, 29].

VAT, a fat depot strongly linked to insulin resistance, T2D and systemic inflammation, has also been associated with cardiac left ventricular hypertrophy and diastolic dysfunction in adults [30]. Here we show that VAT reductions correlated with decreased HFF and CO, and improved insulin sensitivity, paralleling findings in adults and adolescents without T2D undergoing MBS [16, 31]. Further, VAT is known to secrete free fatty acids into the portal vein and circulation, contributing to hepatic steatosis, hepatic and muscle insulin resistance, vascular dysfunction and dyslipidemia [32]. In addition, VAT also releases pro‐inflammatory cytokines such as tumour necrosis factor‐alpha (TNF‐α) and interleukin‐6 (IL‐6), both of which are implicated in insulin resistance and endothelial dysfunction [33]. Although prior studies have reported reductions in VAT and SAT following VSG in young people without T2D using methods other than MRI [34], our use of MRI provides a more precise and anatomically resolved quantification of abdominal fat compartments. Thus, the reductions in VAT we report may have contributed to reducing hepatic steatosis, systemic inflammation and dyslipidemia, and improving multi‐tissue insulin sensitivity and vascular function.

EAT has a unique location surrounding the myocardium and shares direct microcirculation with the heart, and thus may allow for local cardiac, metabolic and inflammatory interactions not seen with other VAT depots. In support, prior evidence in adults links increased EAT volume with CVD [35], and shows higher EAT in adults with T2D versus those who are equally obese without T2D [36]. Limited data suggest that EAT is elevated in adolescents with obesity (but without T2D) compared to lean peers [37]. In these adolescents, EAT has been shown to correlate with several cardiometabolic risk markers including BMI, waist circumference, fasting glucose, insulin, HOMA‐IR and triglycerides. Additional associations have been reported with LV thickness and mass, carotid intima‐media thickness, blood pressure, uric acid, HDL‐C, apolipoprotein B and ALT in youth with obesity and features of the metabolic syndrome [37, 38]. However, these studies used echocardiography, not MRI, which is considered the gold standard method for measuring EAT [37]. While echocardiography is more readily available, it is less sensitive and specific and more susceptible to artefacts related to obesity and therefore is a less precise method for measuring EAT [39]. Moreover, EAT and PAT volume in youth with T2D are completely unexplored despite clear evidence of early CVD in this population. We show that significant decreases in EAT and PAT volume are detectable by 3 months post‐VSG and remain reduced at 12 months in youth with T2D. In addition to the changes in cardiac fat depots, we observed meaningful improvements in cardiovascular health and specifically cardiac morphology and function following VSG. This included lower circulating triglycerides, total cholesterol, LDL‐C, SBP and higher HDL‐C, which are consistent with the literature, as well as novel cardiovascular changes including declines in LV mass, RHR, MAP and LVCO, aspects not previously examined in youth with T2D. While prior studies in adults have demonstrated associations between PAT/EAT and cardiometabolic risk factors, including insulin resistance and LV mass, we did not observe significant correlations between changes in PAT or EAT and changes in these outcomes in our cohort. Nonetheless, the reductions in ectopic fat depots remain important findings, and may reflect early remodelling processes that may precede measurable changes in these clinical markers.

Previous research demonstrated that EAT contributes to pericardial restraint, the force of the pericardium squeezing the heart, which can reduce cardiac output and EF, and may play a direct role in ventricular remodelling [31]. Given that EAT has been linked to adverse cardiac and vascular remodelling [40], the EAT reduction we observed may have potentially contributed to the observed reduction in LV mass, RHR, SBP and MAP, possibly due to both decreased mechanical compression and/or improved myocardial metabolism. As EAT is strongly associated with inflammation in the setting of obesity and diabetes [41], the reduction in EAT observed here may have also decreased inflammation, and subsequently improved insulin sensitivity [42]. Although the reduction in cardiac fat depots did not correlate with the improvement in HOMA‐IR as shown in adults or with the other cardiovascular measures we assessed, the amounts of baseline PAT and EAT in our youth were much lower than previously reported in adults with T2D (~170 cm^3^ vs. our ~49cm^3^) [36] and/or we may have been underpowered for these correlations. Schusterova et al. also did not find a significant correlation in HOMA‐IR and EAT in children with obesity; however, they used echocardiography rather than MRI [37]. In a separate study that utilised MRI to measure EAT, adolescents with obesity had EAT volumes that were similar to those observed in our cohort, and higher EAT volume compared to lean controls; however similar to our findings, no significant correlation was found between EAT volume and HOMA‐IR [43].

The decline in CO we observed is likely not an unhealthy finding; rather, it is an expected response to reduced total metabolic demand, circulating blood volume and fat and lean mass tissue. This pattern is consistent with prior adult literature, where MBS leads to early reductions in CO and LVEDV followed by stabilisation as the body adapts to a lower metabolic workload [44], as well as improvements in LV mass and cardiac strain [45], but it is novel in youth. Since we saw no change in SV post‐operatively, the significant decline in RHR we observed was also likely a key driver of reduced CO, pointing to autonomic dysfunction as an important area for future study. Notably, improvements in insulin sensitivity were correlated with reductions in RHR and CO, suggesting that these metabolic improvements are accompanied by favourable cardiovascular adaptations. Other potential mechanisms underlying these cardiac changes may involve incretins with cardiovascular targets like GLP‐1, or improvements in vascular stiffness or endothelial function, additional areas for further study [46].

### 4.3 Body Composition and Lean Mass

Few studies have looked specifically at the impact of MBS on body composition such as FM and FFM, and not all have evaluated VSG. Our findings of significant FM loss accompanied by more modest lean mass loss mirror results from other adolescent VSG studies. For example, one study found that 12 months post‐VSG, adolescents experienced weight loss of 36.2 kg (27% weight loss), resulting in an 8% reduction in percent body fat, a 25 kg decrease in absolute FM, a 10.8 kg loss in lean mass, and a 0.85 kg reduction in VAT mass [47]. In a similar study 12 months after VSG, youth experienced a total weight loss of 47.8 kg (34.6% weight loss), with reductions in FM and FFM of 39.7 and 6.7 kg, respectively [48]. Although both studies included youth undergoing VSG and saw slightly larger changes in body composition than we report, they lacked any participants with T2D and utilised different methods for measuring body composition than the air‐displacement plethysmography we utilised. Notably, however, across all of these studies, including our own, the proportion of FM lost consistently exceeded that of lean mass, underscoring the beneficial effect of preferential mobilisation of adipose tissue following VSG. This is especially important in youth with T2D, for whom preservation of FFM plays a key role in maintaining glucose disposal and insulin sensitivity.

### 4.4 Study Limitations and Strengths

Our study has limitations that should be acknowledged. First, the follow‐up period of 12 months does not allow for assessment of the longer‐term durability of the changes seen. Weight loss and metabolic adaptations can continue to evolve beyond the first postoperative year, including in individuals who experience weight regain. Therefore, future studies should extend follow‐up to determine whether early fat redistribution predicts sustained cardiometabolic improvements. Additionally, research should explore the impact of weight regain on body composition and cardiometabolic outcomes, particularly regarding fat re‐deposition patterns. Second, we did not specifically assess inflammatory markers beyond liver transaminases. Future studies incorporating systemic and tissue‐specific inflammatory profiling could help clarify the role of adipose tissue inflammation in post‐surgical metabolic adaptation. Additionally, the small sample size was likely underpowered for some outcomes and did not allow for subgroup analyses. Additionally, the lack of control groups such as youth with obesity without T2D or those undergoing non‐surgical interventions, limits our ability to attribute observed changes specifically to VSG. While these results provide important exploratory data, larger controlled studies are necessary to confirm these findings and clarify mechanisms.

Our study also has several strengths that should be highlighted. The use of gold‐standard MRI to precisely quantify multiple adipose tissue depots in a longitudinal design, particularly HFF, VAT, EAT and PAT, which are underexplored but highly prevalent in youth with T2D, provides novel insights into baseline ectopic fat deposition and into the remarkable fat distribution changes after MBS. Our assessment of ectopic fat, body composition and cardiovascular morphology and function also goes beyond the simple clinical outcomes reported in most adolescent MBS studies. Further, to our knowledge, this is the first study to show that EAT and PAT are modifiable with VSG in youth, factors that may contribute to the early CVD events seen in youth‐onset T2D [49], and the first to use the gold‐standard method of MRI in youth with T2D. Additionally, our study exclusively examined adolescents with T2D, and focused on VSG, adding to the gaps in the adolescent MBS literature in this high‐risk population. Furthermore, the study's prospective design, assessments controlled for physical activity and performed in the fasting state, and comprehensive longitudinal assessments at three and 12 months allowed for assessment of both early and longer‐term metabolic adaptations, helping to clarify the timeline of fat loss and cardiometabolic improvements.

## 5 Conclusions and Future Directions

The findings from the IMPROVE‐T2D study demonstrate that VSG leads to rapid and sustained reductions in pro‐inflammatory ectopic fat depots, including HFF, VAT, EAT and PAT, alongside significant improvements in weight, SAT, insulin sensitivity, lipid profiles, SBP, MAP, RHR, LV mass and CO in youth with T2D, all of which remained significant while controlling for weight loss. These results reinforce the potential of MBS as an unprecedentedly powerful intervention for high‐risk youth, far beyond the effects of any other interventions studied in youth with T2D to date. These findings also highlight the important linkage between fat distribution and the early metabolic and cardiovascular improvements that occur after MBS, specifically liver and visceral fat, which correlated strongly with improvements in insulin sensitivity, and cardiac morphology and function. Moreover, our data and others show the likely beneficial outcome of higher loss of fat versus lean mass with MBS. Longer‐term future studies should aim to determine whether baseline or early changes in these measurable fat depots predict longer‐term cardiometabolic outcomes, as well as underlying mechanisms, and comparisons of the outcomes of MBS to newer pharmacologic agents. As the prevalence of youth‐onset T2D continues to rise, these results underscore the benefit of early, aggressive treatment strategies to mitigate long‐term cardiometabolic risk, as well as the importance of including criteria beyond weight, both for deciding who should be considered for surgery, and for determining surgical success.

## Author Contributions

T.J.D. wrote and edited the manuscript, performed research procedures and conducted parts of the analyses. L.G., B.F. and K.S.H. performed cardiac function analysis. T.J.D., H.H.H., B.F. and L.B. performed the adipose tissue analyses. M.G.C. reviewed and edited the manuscript and assisted in the analyses. L.P. performed the statistical analysis and contributed to the methods. M.M.K., D.W., J.L.H.‐D., J.E.B.R. and T.H.I. reviewed and edited the manuscript. P.B. and K.J.N. designed the study, performed research procedures, reviewed and edited the manuscript.

## Conflicts of Interest

P.B. reports serving or having served as a consultant for AstraZeneca, Bayer, Bristol‐Myers Squibb, Boehringer Ingelheim, Eli‐Lilly, L.G. Chemistry, Sanofi, Novo Nordisk and Horizon Pharma. P.B. also serves or has served on the advisory boards and/or steering committees of AstraZeneca, Bayer, Boehringer Ingelheim, Novo Nordisk and XORTX. M.G.C. serves as site PI for Eli‐Lilly and Amino CO and consulting for Novo Nordisk and Eli Lilly. T.H.I. has received consulting fees from Standard Bariatrics, Teleflex, Medtronic, Mediflix, Independent Medical Expert Consulting Services and royalties from Wolters Kluwer (UpToDate) all unrelated to this project. M.M.K. is a site PI for clinical trials sponsored by Rhythm Pharmaceuticals.

## Data Availability

The data that support the findings of this study are available from the corresponding author upon reasonable request.
